# Urinary and Serum Concentration of Deoxynivalenol (DON) and DON Metabolites as an Indicator of DON Contamination in Swine Diets

**DOI:** 10.3390/toxins15020120

**Published:** 2023-02-02

**Authors:** Josiane C. Panisson, Michael O. Wellington, Michael A. Bosompem, Veronika Nagl, Heidi E. Schwartz-Zimmermann, Daniel A. Columbus

**Affiliations:** 1 Prairie Swine Centre Inc., Saskatoon, SK S7H 5N9, Canada; 2 Department of Animal and Poultry Science, University of Saskatchewan, Saskatoon, SK S7N 5A8, Canada; 3 DSM-BIOMIN Research Centre, Technopark 1, 3430 Tulln, Austria; 4 Institute of Bioanalytics and Agro-Metabolomics, Department of Agrobiotechnology (IFA-Tulln), University of Natural Resources and Life Sciences Vienna, 3430 Tulln, Austria

**Keywords:** biomarkers, deoxynivalenol, iso-DON, DOM, metabolites, serum, urine

## Abstract

Pig health is impaired and growth performance is reduced when exposed to deoxynivalenol (DON). The measurement of DON in individual feedstuffs and complete swine diets is variable because of the inconsistent distribution of mycotoxins in feed and the difficulties in obtaining representative samples. We investigated whether measuring DON and its metabolites in biological samples could be used as a predictor of DON ingestion by pigs. Blood samples were collected between 3 and 4 h after the morning meal and urine samples were quantitatively collected over a 24 h period on d 40 and 82 of the study to evaluate serum and urinary content of DON and DON metabolites (iso-deoxynivalenol, DON-3-glucuronide, DON-15-glcurunide, deepoxy-deoxynivalenol, iso-deepoxy-deoxynivalenol, deepoxy-deoxynivalenol-3-glucuronide, and deepoxy-deoxynivalenol-15-glucuronide). The intake of DON was positively correlated with urinary DON output. Similarly, there was an increase in serum DON level with increasing DON intake. Overall, it was found that DON intake correlated with DON concentration in urine and blood serum when samples were collected under controlled conditions. Analyzing DON levels in urine and blood serum could be used to predict a pig’s DON intake.

## 1. Introduction

Deoxynivalenol (DON) is one of the most prevalent mycotoxins produced by *Fusarium* spp. Deoxynivalenol commonly contaminates cereal grains, including wheat, barley, and corn. The DON contamination of feed ingredients and complete feeds has been shown to have negative effects on swine, including reduced growth performance, decreased nutrient utilization, and impaired health [1,2,3]. There are notable differences in the susceptibility to DON among animal species, likely due to differences in the metabolism of DON, with pigs considered highly sensitive to DON exposure [4,5]. Reduced feed intake, digestion disorders (e.g., gastroenteritis, lesions of the gastrointestinal tract, and reduced nutrient absorption), immune suppression, and reduced growth performance are some of the typical negative effects of mycotoxin exposure [3,6,7,8]. In addition to reduced feed intake and growth performance, consuming feed contaminated with DON results in damage to the epithelial cells of the intestinal tract and altered intestinal growth and barrier function as well as increased susceptibility to enteric pathogen challenge [9,10]. Damage to the intestine also results in a reduction in nutrient absorption [10] and, therefore, availability for growth. Once absorbed, DON inhibits protein synthesis, causes kidney and liver damage, and can suppress immune function, resulting in a decreased ability to resist disease challenge [11]. Deoxynivalenol has also been shown to damage the intestinal tract due to direct toxicity [7].

The determination of the concentration of mycotoxins, including DON, in feedstuffs and complete feeds is required in order to assess risk. However, the accurate determination of DON continues to be a challenge, largely due to the inconsistent distribution of DON within batches of feedstuffs and complete feeds. While DON intake has been shown to affect the performance and health of pigs, the response to DON intake can also be variable and negative effects may not be evident at low levels (e.g., <1 mg/kg) [1,2]. The quantification of DON and its metabolites in biological samples may be a more accurate method of determining actual DON intake in pigs [12]. Therefore, the objective of this study was to investigate the analysis of DON and its metabolites in biological samples as an indicator of DON ingestion in pigs.

## 2. Results and Discussion

Several studies have shown the negative effects of DON exposure on pig performance and health [1,2,13,14]. Once absorbed, DON can cause kidney, liver, and immune system damage, resulting in decreased resistance to disease [15]. Considering the difficulties in measuring DON in feedstuffs and complete feed, our objective was to determine whether the content of DON and its metabolites in biological samples could represent actual DON intake in pigs. Previous work has demonstrated a high correlation between DON consumption and DON presence in the body (urine and blood) [2,12]. However, to the best of our knowledge, this is the first combined direct quantification of DON and its metabolites iso-deoxynivalenol (isoDON), DON-3-glucuronide (DON-3-GlcA), DON-15-glcurunide (DON-15-GlcA), deepoxy-deoxynivalenol (DOM), iso-deepoxy-deoxynivalenol (isoDOM), DOM-3-glucuronide (DOM-3-GlcA), and DOM-15-glucuronide (DOM-15-GlcA) in biological specimens.

**Figure 1:**
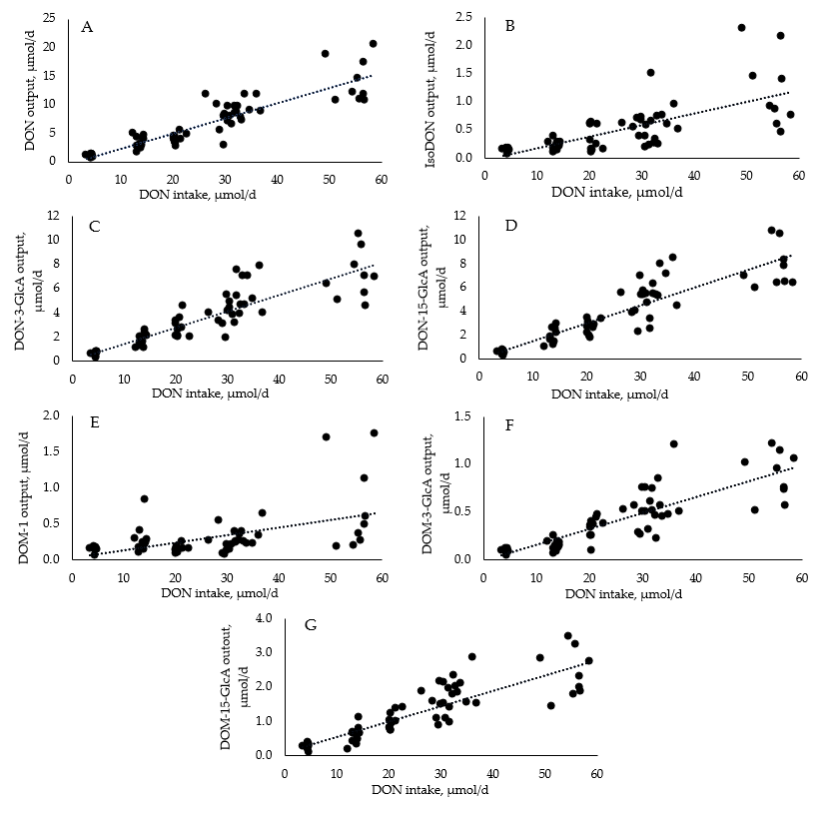
Regression relationship between DON intake and the excretion of DON (**A**) and its metabolites (isoDON, (**B**); DON-3-GlcA, (**C**); DON-15-GlcA, (**D**); DOM-1, (**E**); DOM-3-GlcA, (**F**); DOM-15-GlcA, (**G**)) in urine over a 24 h period in pigs receiving DON-contaminated diets (1, 3 or 5 mg/kg). Points in the graph represent combined pig data from two time points [day 40 (grower phase) and day 82 (finisher phase); n = 10/treatment per time point]. Data are expressed as daily DON intake (μmol/d) and urinary excretion of DON and its metabolites (μmol/d). The equation and the coefficient of determination (*R*^2^) of the regression curve are for DON: y = 0.2667x − 0.339, *R*^2^ = 0.81 at *p* < 0.001; DOM-1: y = 0.0107x + 0.0337, *R*^2^ = 0.27 at *p* < 0.001; isoDON: y = 0.0208x − 0.0288, *R*^2^ = 0.48 at *p* < 0.001; DON-3-GlcA: y = 0.1343x + 0.1302, *R*^2^ = 0.75 at *p* < 0.001; DON-15-GlcA: y = 0.1499x − 0.0032, *R*^2^ = 0.79 at *p* < 0.001; DOM-3-GlcA: y = 0.0168x − 0.0113, *R*^2^ = 0.71 at *p* < 0.001; and DOM-15-GlcA: y = 0.0448x + 0.112, *R*^2^ = 0.73 at *p* < 0.001.

Linear regression modelling indicated a positive correlation between DON intake and the urinary excretion of DON and DON metabolites (Figure 1). In general, a dose-dependent increase in urinary DON excretion was observed with increasing DON intake. Overall, the total DON recovered in urine (sum of DON and metabolites) accounted for 63.5% of the DON intake. This proportion falls within the wide range of previously reported recovery rates of 27.9, 44.0, and 84.8% [16,17,18,19].

Descriptively, there was little difference between the recovery of DON in grower (61.5%, all groups) and finisher pigs (65.6%). This is interesting, as previous studies have suggested that the bioavailability of DON increases after prolonged exposure [20], which was not substantiated by our data. This may be due to a number of factors, including the duration of study, the amount and source of DON in diets, and the timing of sample collection.

The quantitative recovery of DON and its metabolites in urine allows for an estimation of the toxin’s bioavailability in pigs [20]. A significant proportion of urinary DON was found in the conjugated form, with only a minor proportion of the ingested DON converted to DOM, which was subsequently conjugated to DOM-GlcAs. The major metabolites in urine were DON (24.7% of intake recovered), DON-15-GlcA (14.5%), and DON-3-GlcA (13.9%), followed by DOM-15-GlcA (5.1%), IsoDON (2.1%), DOM-3-GlcA (1.6%), and DOM (1.5%).

**Figure 2:**
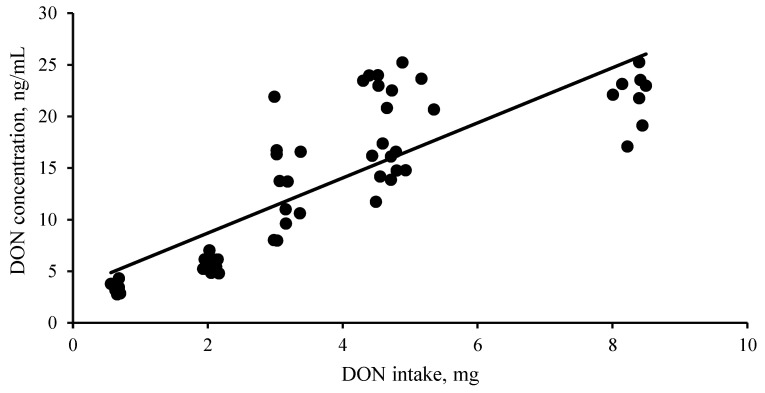
Regression relationship between DON intake and DON concentration in serum after a single meal. The blood samples were obtained within 3 and 4 h after pigs received a DON-contaminated diet (1, 3, or 5 mg/kg). Points in the graph represent combined pig data from two time points [day 40 (grower phase) and day 82 (finisher phase); n = 10/treatment per time point]. Data are expressed as DON intake from a single meal and the serum DON concentration (ng/mL). The equation and the coefficient of determination (*R*^2^) of the regression curve are y = 1.45x + 2.1, *R*^2^ = 0.77 at *p* < 0.05.

There was a positive correlation between DON intake in a single meal and DON concentration in blood serum (Figure 2). Absorption of DON in pigs is rapid, with peak serum concentrations occurring between 1.5 h and 4 h after intake [18,20]. The lack of DOM in serum is likely due to DON absorption primarily occurring in the proximal small intestine, whereas DON de-epoxidation occurs mainly in the large intestine.

## 3. Conclusions

The results indicate that DON intake and concentration of DON in blood serum and DON and DON metabolite concentration in urine were highly correlated. Moreover, the recovery of total DON in urine remained consistent across pig age and DON intake. Overall, the analysis of DON and DON metabolites in biological samples may be an accurate method of determining DON intake in pigs under controlled sampling conditions.

## 4. Materials and Methods

### 4.1. Animals, Housing, Diets, and Experimental Design

Information on the experimental procedure and performance data has been published previously [2]. In summary, a total of 90 male and 90 female pigs of a commercial lineage (Camborough Plus x C337; PIC, Winnipeg, Manitoba, Canada) were selected, with a mean initial body weight of 35.9 ± 1.1 kg. The pigs were housed in a mixed sex group (6 pigs/pen) and randomly assigned to 1 of 3 dietary treatments for 82 d in an environmentally controlled room. The dietary treatments were graded levels of DON (1, 3, or 5 mg/kg), fed for a period of 82 d. On d 35 and 77 of the study, 1 representative pig/pen was selected and placed in a metabolism crate and fed daily at 2.8 × maintenance metabolizable energy requirement (197 kcal/kg BW^0.60^; [21]) in 2 equal meals at 07:00 h and 15:00 h. Urine samples were collected for a 24-h period on d 40 and 82 of the study. Blood samples were also collected on the same days between 3 and 4 h after the morning meal. Blood samples were collected into 10 mL additive-free vacutainer tubes (BD vacutainer, Mississauga, ON, Canada), centrifuged at 2500× *g* for 15 min, and serum sub-sampled and stored at −20 °C until further analysis.

### 4.2. Analysis of DON and DON Metabolites

Mycotoxin analysis was performed at the laboratory of the DSM-BIOMIN Research Center (Tulln, Austria). Analytical standards for DON and DOM-1 were acquired commercially (Romer Labs GmbH, Tulln, Austria), whereas the standard for DON-3-GlcA was produced by chemical synthesis [22]. Analytical standards of DOM-3-GlcA, DOM-15-GlcA, isoDON, and isoDOM were produced as described in [16]. Phosphate buffered saline (PBS) was obtained from Sigma–Aldrich (Vienna, Austria), methanol (MeOH) and acetic acid from VWR International (Vienna, Austria), and acetonitrile from Chem-Lab NV (Zedelgem, Belgium). In urine, direct quantification of DON, DON-3-GlcA, isoDON, DOM-1, DOM-3-GlcA, DOM-15-GlcA, and isoDOM was performed. DON-15-GlcA was quantified via the DON-3-GlcA standard. Analysis determination was performed on a QTRAP 6500 using a previously described HPLC-MS/MS method [16]. All analyses were conducted in duplicate. IsoDOM was not detected in any of the samples, and therefore not included in further data evaluation.

Serum samples collected from pigs were subjected to enzyme pretreatment prior to analysis determination. For this purpose, 35 mg of β-glucuronidase (Escherichia coli, type IX-A; Sigma–Aldrich, Oakville, Canada) was dissolved in 2.5 ml of PBS and added to 50 μL for every 100 μL of serum. Following incubation for 18 h (37 °C, 80 rpm), 300 μL of MeOH/acetic acid (99.8/0.2, *v*/*v*) was added. The HPLC-MS/MS assay was conducted as described for the urine samples.

### 4.3. Statistical Analysis

All data were verified for normality using the PROC UNIVARIATE (SAS Institute, Cary, NC, USA, Version 9.4), and studentized residual analyses were used to identify outliers. The relationship between DON intake and urinary output of DON and its metabolites and DON content in serum were analyzed using regression (PROC REG, SAS Institute, Version 9.4). Descriptive statistics were also used for urine and serum metabolites. Differences between means were considered significant at *p* ≤ 0.05.

## Data Availability

All data available upon reasonable request to the corresponding author.
